# Mosaic attenuation in non-fibrotic areas as a predictor of non-usual interstitial pneumonia pathologic diagnosis

**DOI:** 10.1038/s41598-022-10750-7

**Published:** 2022-05-04

**Authors:** Ignacio Gayá García-Manso, Juan Arenas-Jiménez, Raquel García-Sevila, Sandra Ruiz-Alcaraz, Marina Sirera-Matilla, Elena García-Garrigós, María Ángeles Martínez-García, Luis Hernández-Blasco

**Affiliations:** 1 Department of Pulmonology, ISABIAL, Hospital General Universitario Dr. Balmis, Pintor Baeza, 11, 03010 Alicante, Spain; 2 Department of Radiology, ISABIAL, Hospital General Universitario Dr. Balmis, Alicante, Spain; 3 Department of Pulmonology, ISABIAL, Hospital General Universitario de Elche, Alicante, Spain; 4 Department of Clinical Medicine, UMH, Alicante, Spain

**Keywords:** Medical research, Respiratory tract diseases

## Abstract

The new radiological diagnostic criteria for diagnosing idiopathic pulmonary fibrosis (IPF) seek to optimize the indications for surgical lung biopsy (SLB). We applied the new criteria to a retrospective series of patients with interstitial lung disease (ILD) who underwent SLB in order to analyse the correlation between the radiological findings suggestive of another diagnosis (especially mosaic attenuation and its location with respect to fibrotic areas) and the usual interstitial pneumonia (UIP) pathologic diagnosis. Two thoracic radiologists reviewed the HRCT images of 83 patients with ILD and SLB, describing the radiological findings and patterns based on the new criteria. The association of each radiological finding with radiological patterns and histology was analysed. Mosaic attenuation is highly prevalent in both the UIP and non-UIP pathologic diagnosis and with similar frequency (80.0% vs. 78.6%). However, the presence of significant mosaic attenuation (≥ 3 lobes) only in non-fibrotic areas was observed in 60.7% of non-UIP pathologic diagnosis compared to 20.0% in UIP. This finding was associated with other diagnoses different from IPF, mostly connective tissue disease-associated interstitial lung disease (CTD-ILD) and hypersensitivity pneumonitis (HP). In our series of pathologically confirmed ILD, mosaic attenuation in non-fibrotic areas was a predictor of non-UIP pathologic diagnosis, and was associated with other diagnoses different from UIP, mostly CTD-ILD and HP. If confirmed in larger series, this finding could constitute a valuable tool for improving the interpretation of radiological patterns.

## Introduction

**Figure 1:**
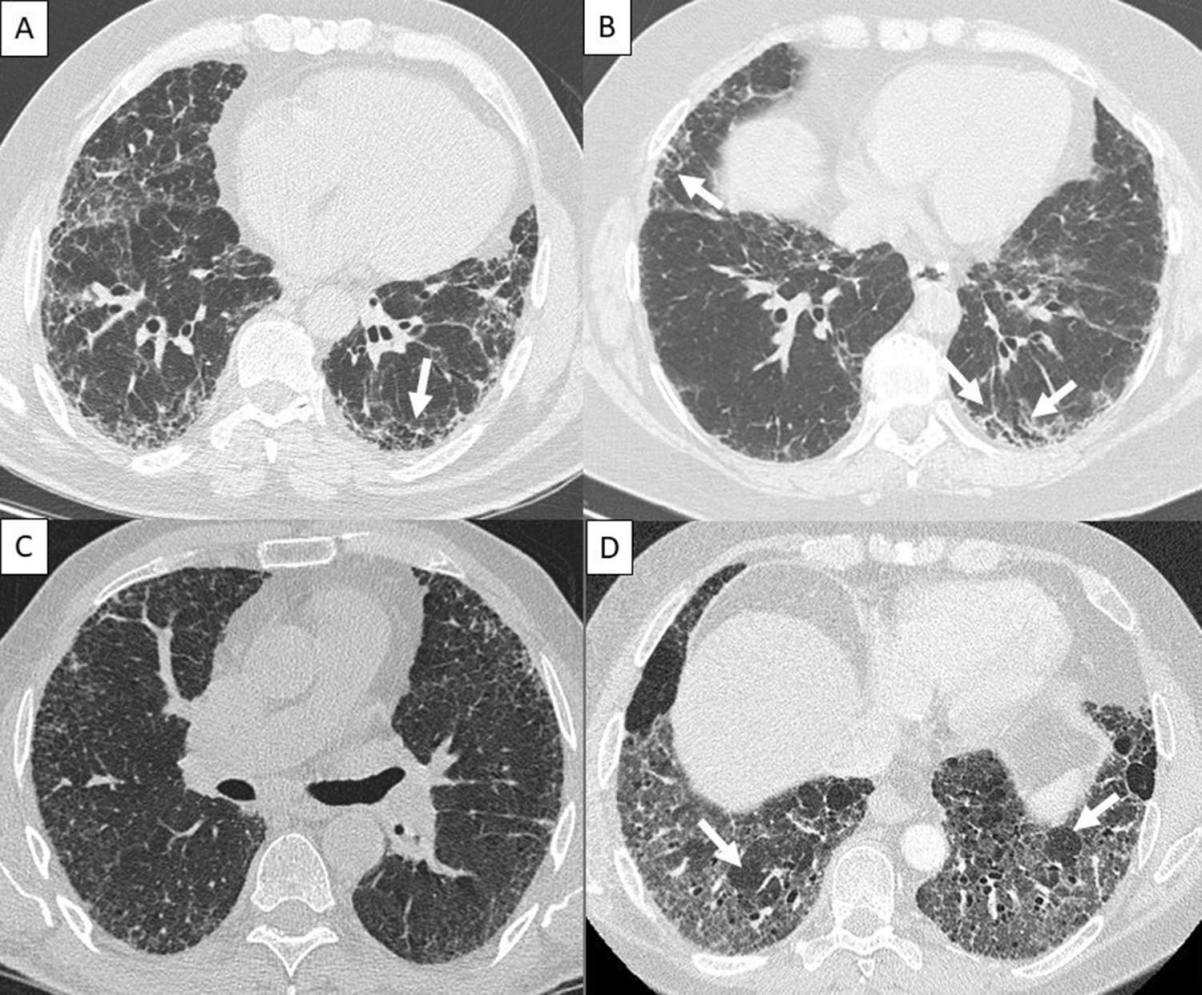
High-resolution computed tomography images of radiological patterns. (**A**) 67-year-old man with idiopathic pulmonary fibrosis (IPF) and usual interstitial pneumonia (UIP) pathological diagnosis. UIP radiological pattern with subpleural peripheral reticulation, traction bronchiectasis and honeycombing (arrows). (**B**) 66-year-old woman with IPF and UIP pathological diagnosis. Probable UIP radiological pattern with subpleural peripheral reticulation and traction bronchiectasis (arrows), without honeycombing. (**C**) 75-year-old man with IPF and UIP pathological diagnosis. An indeterminate pattern was seen consisting of predominating peripheral reticulation which had a diffuse distribution, shown at this section at the middle lung zone. (**D**) 48-year-old man with IPF and UIP pathological diagnosis. Non-UIP radiological pattern with ground-glass opacities and mosaic attenuation within reticulation areas.

The radiological criteria for the diagnosis of idiopathic pulmonary fibrosis (IPF) have recently been updated. First, a new edition of joint clinical practice guidelines for diagnosing IPF has been published by the American Thoracic Society (ATS), European Respiratory Society (ERS), Japanese Respiratory Society (JRS), and Latin American Thoracic Association (ALAT)^1^, while the Fleischner Society has also led the development of an expert consensus paper for diagnostic criteria^2^. Although there are some small differences between the new guidelines^1,2^, both describe, for the first time, four radiological patterns: “usual interstitial pneumonia (UIP),” “probable UIP,” “indeterminate for UIP,” and either “alternative diagnosis”^1^ or “CT features most consistent with non-IPF diagnosis”^2^ (hereafter, “non-UIP”) (Fig. 1).

Since the publication of the older version of the guidelines in 2011^3^, the literature has increasingly supported the tendency to trust the high-resolution computed tomography (HRCT) findings. The new radiological criteria have been improving the diagnostic accuracy until reaching the current version of the guidelines, with limited indications for obtaining histological samples. However, the role of some of the radiological findings described by the guidelines as suggestive of another diagnosis different from UIP has not been fully clarified.

Mosaic attenuation is among these findings suggestive of another diagnosis, due to its closer association with other diagnoses, mainly hypersensitivity pneumonitis (HP)^4–7^. However, mosaic attenuation is frequent in patients with UIP pathologic diagnosis^8^ or IPF^9^. Different authors have tried to clarify the value of this finding by analysing whether the extent or location of mosaic attenuation can help to better predict a non-UIP pathologic diagnosis or a diagnosis other than IPF.

The Fleischner Society White Paper^2^ states that the presence of mosaic attenuation in non-fibrotic areas could be useful for differentiating between IPF and HP. In usual clinical practice, radiologists may consider whether the location of mosaic attenuation is associated with fibrotic areas or appears far from these areas, surrounded by healthy lung; however, this affirmation is not supported by published evidence, as this relationship has never been confirmed. Moreover, the ATS/ERS/JRS/ALAT guidelines^1^ propose new lines of research like quantitative or qualitative analysis of mosaic attenuation in order to determine how to differentiate UIP in patients with IPF from UIP-like patterns in patients with other diagnoses.

Thus, in our study we decided to retrospectively review HRCTs at the time of diagnosis, applying the new criteria to a series of patients with ILD confirmed by surgical lung biopsy in order to analyse the correlation between the radiological findings suggestive of another diagnosis (especially mosaic attenuation and its location with respect to fibrotic areas) and the UIP pathologic diagnosis. 

## Material and methods

This study was approved by “Ethics Committee for Research with Medicines of the Department of Health—Hospital General Universitario de Alicante” and performed in compliance with the principles of the Declaration of Helsinki.

### Study population

Eligible patients were those with ILD who had undergone surgical lung biopsy from 2007 to 2019 in our centre, a tertiary reference hospital for thoracic surgery in our province. Patients were included if they were diagnosed with incident ILD with suspicion of IPF, and their clinical records contained HRCT images taken within a year of their lung biopsy. Exclusion criteria and data for excluded patients are detailed in the supplementary material.

### HRCT evaluation

For our study, we read the chest CT scans performed at the nearest time point to the moment of diagnosis. Two thoracic radiologists, JAJ (24 years’ experience) and MSM (4 years’ experience), blinded to the patient’s clinical data and diagnosis, independently reviewed the images. Using a PACS workstation, they performed a multiplanar evaluation, having the possibility to read coronal and sagittal planes to improve characterization and quantification. The discrepancies in the qualitative variables were resolved in consensus with a third radiologist, EGG (11 years’ experience). The radiological findings were interpreted according to the latest ATS/ERS/JRS/ALAT guidelines^1^ and the Fleischner Society’s glossary of terms for thoracic imaging^10^. More details regarding the examinations and interpretation of the images are provided in supplementary materials.

### Pathological evaluation

Pathological diagnoses were collected from biopsy samples and reviewed according to the criteria established by current guidelines^1^. For the statistical analysis, we classified UIP and probable UIP as “concordant with UIP”, and grouped the other patterns into “non-UIP patterns”.

### Multidisciplinary diagnosis

A multidisciplinary committee, made up of specialists in pulmonology, radiology, pathology, and rheumatology, established the definitive diagnoses based on clinical, radiological, and pathological criteria.

### Statistical analysis

We used the κ coefficient to evaluate interobserver concordance for the findings and the radiological patterns identified, and Spearman’s correlation coefficient to analyse the variation in the extent of each finding and the degree of affectation. The association between categorical variables was analysed using the chi-squared test. To compare quantitative variables by radiological patterns, we used ANOVA (or the Kruskal–Wallis test if the distribution was not normal). *P* values of less than 0.05 were considered statistically significant. Analyses were performed using the SPSS statistical package (IBM, v.19) for Windows.

### Ethics approval and consent to participate

This study was approved by “Ethics Committee for Research with Medicines of the Department of Health—Hospital General Universitario de Alicante” and performed in compliance with the principles of the Declaration of Helsinki. Informed consent waiver was obtained by “Ethics Committee for Research with Medicines of the Department of Health—Hospital General Universitario de Alicante” because of the retrospective nature of the study and the absence of intervention.

## Results

**Figure 2:**
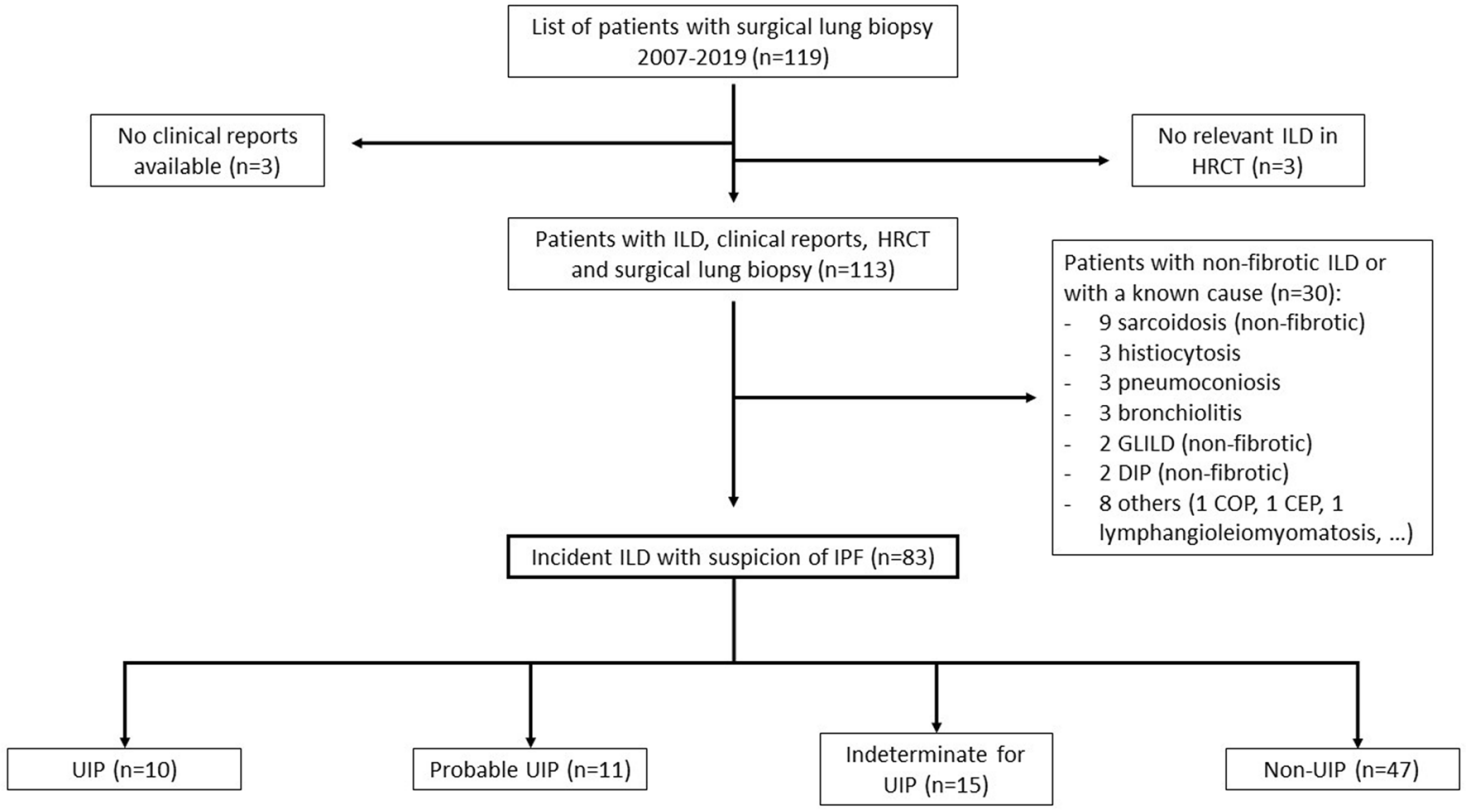
Flow chart. Description of the patients included and excluded in the study. *ILD* interstitial lung disease, *HRCT* high-resolution computed tomography, *GLILD* granulomatous lymphocytic interstitial lung disease, *DIP* desquamative interstitial pneumonia, *COP* cryptogenic organizing pneumonia, *CEP* chronic eosinophilic pneumonia, *IPF* idiopathic pulmonary fibrosis, *UIP* usual interstitial pneumonia.

The final sample was of 83 patients. Figure 2 shows the flow chart for selecting patients in whom the new guidelines were considered applicable. Table 1 presents the population’s baseline characteristics. Mean age at diagnosis was 60.0 ± 11.0 years, and there was a predominance (59.0%) of men. Median time between the included CT and the lung biopsy was 2.9 months (interquartile range 0.9, 4.1). A history of tobacco use was reported in 62.8% of the patients (mean exposure 33.8 ± 24.9 pack-years). Mean forced vital capacity was 2303.9 ± 736.9 mL (68.8% ± 18.6), and mean diffusing capacity of the lung for carbon monoxide was 54.0% ± 20.3.

**Table 1:** Population’s baseline characteristics.

	Total population (n = 83)
**Demographics**	
Age, years	60.0 ± 11.0
Male sex	49 (59.0)
Caucasian	79 (95.2)
ILD’s family history	6 (7.2)
Ever smoked	49 (62.8)
Pack-years	33.8 ± 24.9
Duration of symptoms, months*	6.0 (3.0–12.0)
Time between HRCT and biopsy, months*	2.4 (0.9–4.1)
Crackles	60 (72.3)
Clubbing	14 (16.9)
**Pulmonary function testing**	
FVC, mL	2303.9 ± 736.9
FVC, %	68.8 ± 18.6
FEV1/FVC	85.2 ± 14.9
DLCO, %	54.0 ± 20.3
TLC, mL	3627.7 ± 1019.0
TLC, %	66.6 ± 17.5
6-min walking distance, m	432.8 ± 98.3

Data are presented as n (%) or mean ± SD, except variables marked with *, presented as median (interquartile range).

*ILD* interstitial lung disease, *HRCT* high resolution computed tomography, *FVC* forced vital capacity, *FEV1* forced expiratory volume in 1 s, *DLCO* diffusing capacity of the lung for carbon monoxide, *TLC* total lung capacity.

### Interobserver agreement

The correlation between radiologists is shown in Table 2. It was almost perfect (κ = 0.80–1.00) for the findings of reticulation, traction bronchiectasis, and honeycombing, and substantial (κ = 0.60–0.80) for the presence of ground-glass opacities and the classification of radiological patterns. Regarding mosaic attenuation, agreement was substantial for this finding in at least 3 lobes, located in non-fibrotic areas, its extension and number of lobes, and moderate (κ = 0.40–0.60) for overall mosaic attenuation and within fibrotic areas. Supplementary Table S.1 details the correlation for the pattern classification.

**Table 2:** Interobserver agreement.

	Interobserver agreement (95% CI)
**Radiological findings**	
Reticulation	0.849 (0.634–1.000)^a^
Extent of reticulation	0.832 (0.718–0.908)^b^
Traction bronchiectasis	0.845 (0.671–0.966)^a^
Extent of traction bronchiectasis	0.884 (0.810–0.934)^b^
Honeycombing	0.828 (0.675–0.945)^a^
Extent of honeycombing	0.890 (0.784–0.962)^b^
Ground-glass	0.729 (0.479–0.914)^a^
Ground-glass only within fibrotic areas	0.496 (0.309–0.676)^a^
Ground-glass only in non-fibrotic areas	0.711 (0.474–0.887)^a^
Extent of ground-glass	0.902 (0.847–0.938)^b^
Mosaic attenuation	0.541 (0.372–0.725)^a^
Mosaic attenuation ≥ 3 lobes	0.640 (0.473–0.783)^a^
Mosaic attenuation ≥ 3 lobes only within fibrotic areas	0.546 (0.222–0.802)^a^
Mosaic attenuation ≥ 3 lobes only in non-fibrotic areas	0.610 (0.430–0.764)^a^
Extent of mosaic attenuation	0.767 (0.652–0.851)^b^
Lobes with mosaic attenuation	0.733 (0.586–0.840)^b^
Cysts	0.730 (0.461–0.917)^a^
Emphysema	0.891 (0.737–1.000)^a^
Consolidation	0.628 (0.295–0.849)^a^
Extent of consolidation	0.670 (0.321–0.881)^b^
Nodules	0.659 (0.349–0.881)^a^
Lymph nodes	0.583 (0.157–0.886)^a^
Overall extent of fibrosis	0.703 (0.573–0.800)^b^
**Distribution**	
Peripheral predominance	0.689 (0.498–0.848)^a^
Peribronchovascular predominance	0.657 (0.485–0.807)^a^
Basal predominance	0.641 (0.467–0.802)^a^
**Radiological pattern**	0.633 (0.493–0.759)^a^

All correlation results are statistically significant, *P* values < 0.001.

^a^Interobserver correlation shown as κ coefficient for qualitative variables.

^b^Interobserver correlation shown as Spearman’s ρ for quantitative variables.

### Findings by radiological pattern

Table 3 shows the relationship between ground-glass and mosaic attenuation and the patterns identified. Differences were not observed for the total patients with mosaic attenuation or ground-glass opacities, but they were when we analysed their relationship to fibrotic areas. The relationship between the rest of radiologic findings and radiological patterns is shown in Supplementary Table S.2. As expected, honeycombing appeared in all patients with the UIP pattern, and findings suggestive of another diagnosis different from UIP appear more frequently in indeterminate and non-UIP patterns.

**Table 3:** Relation between ground-glass and mosaic attenuation and radiological patterns on CT scan.

	UIP (n = 10)	Probable UIP (n = 11)	Indeterminate for UIP (n = 15)	Non-UIP (n = 47)	*P* value
Ground-glass	8 (80.0)	8 (72.7)	13 (86.7)	44 (93.6)	0.217
Ground-glass only within fibrotic areas	7 (70.0)	6 (54.5)	5 (33.3)	9 (19.1)	**0.005**
Ground-glass only in non-fibrotic areas	0 (0.0)	0 (0.0)	1 (6.7)	14 (29.8)	**0.016**
Extent of ground-glass, %	6.3 (1.9–18.1)	7.5 (0.0–12.5)	12.5 (7.5–30.0)	55.0 (25.0–80.0)	**< 0.001**
Mosaic attenuation	8 (80.0)	6 (54.5)	14 (93.3)	38 (80.9)	0.111
Mosaic attenuation ≥ 3 lobes	5 (50.0)	5 (45.5)	6 (40.0)	32 (68.1)	0.177
Mosaic attenuation ≥ 3 lobes only within fibrotic areas	4 (40.0)	3 (27.3)	1 (6.7)	3 (6.4)	**0.014**
Mosaic attenuation ≥ 3 lobes only in non-fibrotic areas	0 (0.0)	1 (9.1)	6 (40.0)	21 (44.7)	**0.012**
Extent of mosaic attenuation, %	11.3 (3.8–22.5)	5.0 (0.0–30.0)	10.0 (5.0–30.0)	12.5 (5.0–35.0)	0.601
Lobes with mosaic attenuation	3.0 (0.8–5.0)	1.5 (0.0–5.0)	2.0 (1.0–4.5)	3.5 (1.0–5.5)	0.331

Data are presented as n (%) or mean ± SD or median (interquartile range).

*UIP* usual interstitial pneumonia. In bold statistically significant differences.

### Pathological diagnosis by radiological pattern

Table 4 shows the results of the analysis of pathologic diagnosis by radiological pattern. We observed a high correlation between UIP pathologic diagnosis and UIP, probable UIP, and indeterminate for UIP radiological patterns, with significantly lower values for the non-UIP pattern. Supplementary Table S.3 shows the relationship between radiological patterns, pathology, and multidisciplinary diagnosis.

**Table 4:** Relation between radiological pattern and UIP pathologic diagnosis.

	UIP (n = 10)	Probable UIP (n = 11)	Indeterminate for UIP (n = 15)	Non-UIP (n = 47)	*P* value
**Pathologic diagnosis**					
Concordant with UIP	10 (100.0)	9 (81.8)	13 (86.7)	23 (48.9)	**0.002**
Non-UIP patterns	0 (0.0)	2 (18.2)	2 (13.3)	24 (51.1)	**–**

Data are presented as n (%).

*UIP* usual interstitial pneumonia. In bold statistically significant differences.

### Radiological findings suggestive of another diagnosis different from UIP

Table 5 shows the radiological findings suggestive of another diagnosis different from UIP classified by pathologic pattern. The most relevant finding is that mosaic attenuation and ground-glass opacities are highly prevalent in both the UIP and non-UIP pathologic diagnosis and with similar frequency; however, in the UIP pathologic diagnosis they appear within fibrotic areas, while in the non-UIP pathologic diagnosis they appear in non-fibrotic areas. When considered significant mosaic attenuation (≥ 3 lobes) in non-fibrotic areas, it was more frequent in non-UIP (60.7%) compared to UIP (20.0%) pathologic diagnosis. Table 6 shows the diagnoses in these patients, having a clear association of this finding with non-UIP pathologic diagnosis and with other diagnoses different from IPF, mostly connective tissue disease-associated interstitial lung disease (CTD-ILD) and HP.

**Table 5:** Radiological findings suggestive of another diagnosis different from UIP by pathologic diagnosis.

Radiological finding suggestive of another diagnosis different from UIP	UIP pathologic diagnosis (n = 55)	Non-UIP pathologic diagnosis (n = 28)	*P* value
Absence of basal predominance	20 (36.4)	14 (50.0)	0.232
Absence of peripheral predominance	12 (21.8)	14 (50.0)	**0.009**
Ground-glass	48 (87.3)	25 (89.3)	0.790
Ground-glass only within fibrotic areas	24 (43.6)	3 (10.7)	**0.002**
Ground-glass only in non-fibrotic areas	3 (5.5)	12 (42.9)	**< 0.001**
Mosaic attenuation	44 (80.0)	22 (78.6)	0.879
Mosaic attenuation ≥ 3 lobes	30 (54.5)	18 (64.3)	0.396
Mosaic attenuation ≥ 3 lobes only within fibrotic areas	10 (18.2)	1 (3.6)	0.063
Mosaic attenuation ≥ 3 lobes only in non-fibrotic areas	11 (20.0)	17 (60.7)	**< 0.001**
Consolidation	5 (9.1)	7 (25.0)	0.051
Nodules	3 (5.5)	10 (35.7)	**< 0.001**
Cysts	8 (14.5)	5 (17.9)	0.695

Data presented as n (%).

*UIP* usual interstitial pneumonia. In bold statistically significant differences.

**Table 6:** Diagnoses by pathologic pattern in patients with mosaic attenuation ≥ 3 lobes only in non-fibrotic areas.

Pathologic pattern	Multidisciplinary diagnosis
11 Concordant with UIP	9 IPF
	2 HP
17 Non-UIP	4 HP
	4 Idiopathic NSIP
	4 CTD-ILD
	2 IPAF
	1 IPF
	1 Idiopathic bronchiolocentric interstitial pneumonia
	1 Unclassifiable ILD

*UIP* usual interstitial pneumonia, *IPF* idiopathic pulmonary fibrosis, *HP* hypersensitivity pneumonitis, *NSIP* nonspecific interstitial pneumonia, *CTD-ILD* connective tissue disease-associated interstitial lung disease, *IPAF* interstitial pneumonia with autoimmune features, *ILD* interstitial lung disease.

## Discussion

We performed a retrospective study in a series of patients with ILD and surgical biopsy in order to analyse the correlation between the radiological findings suggestive of another diagnosis (especially mosaic attenuation and its location with respect to fibrotic areas) and the UIP pathologic diagnosis. We observed that despite mosaic attenuation being a frequent finding in patients with UIP pathologic diagnosis, its appearance exclusively in non-fibrotic areas is suggestive of a non-UIP pathologic diagnosis and is associated with other diagnoses different from IPF.

Some radiological features are defined by the guidelines as “findings suggestive of another diagnosis”^1^ or “CT features more consistent with non-IPF diagnosis”^2^ due to their closer association with other ILDs, as demonstrated in several studies. These features include cysts, marked or extensive mosaic attenuation, predominant ground-glass opacities, nodules or consolidation, and also peribronchovascular predominance with subpleural sparing or upper-lung or middle-lung predominant fibrosis.

Regarding air trapping or mosaic attenuation, although they may be normal findings present in a significant proportion of healthy individuals^11^, they are very frequent in patients with sarcoidosis^12,13^ and have been found to be more closely associated with CTD-ILD than with IPF^14^, with no apparent differences among various CTD^15^. Above all, air trapping and mosaic attenuation are associated with HP^4–7^ and are considered relevant findings in the radiological pattern typical of this pathology^16^. However, these findings are also present in 12.6%^17^ to 21.3%^12^ of patients with UIP pathologic diagnosis. Other series describe them in 35%^6^, 45%^18^, or 51%^9^ of patients diagnosed with IPF, albeit some of these diagnoses could correspond to misdiagnosed HP (up to 40% of them, according to Morell et al.’s series^19^). In our series, up to 80% of the patients with UIP pathologic diagnosis presented some degree of mosaic attenuation, so its presence does not appear sufficient to indicate that this finding is “suggestive of another diagnosis” or to rule out UIP.

One consideration with regard to this finding is the different terminology used. “Air trapping” is often used when areas of diminished attenuation appear on expiratory CT scans, while the concept of “mosaic attenuation” is cited in inspiratory CTs. But some studies use different criteria or use these terms synonymously. Even the very definitions used in the guidelines to consider mosaic attenuation as a finding suggestive of another diagnosis are vague and somewhat subjective, using qualifiers such as “marked”^1^ or “extensive”^2^.

When analysing the extent of the mosaic attenuation to determine its significance, the most frequently used threshold is the involvement of 3 or more lobes, the same cutoff used in the 2011 guidelines^3^. Barnett et al.^9^ tested three different thresholds for the extent of the mosaic attenuation, based on the number of lobes affected. The authors concluded that even though enlarging the extension required to consider a diagnosis of HP increased specificity, no threshold could completely exclude the diagnosis of IPF. In our study, we also analysed the overall extent and the number of lobes affected, without finding differences that could predict UIP pathologic diagnosis. Nevertheless, we decided to consider mosaic attenuation as significant when it affected at least three lobes, as this was the threshold in the previous guidelines and the one with the strongest evidence base in the literature.

Although the Fleischner Society White Paper^2^ suggests that the presence of mosaic attenuation in non-fibrotic areas could be a discriminator between IPF and HP, to our knowledge, such analysis has not been previously performed on a pathologically confirmed series. In our study, we analysed the location of the mosaic attenuation in relation to fibrotic areas, and found that it appeared within fibrotic areas in UIP pathological diagnosis and in non-fibrotic areas in the case of non-UIP pathological diagnosis. We found that significant mosaic attenuation affecting ≥ 3 lobes only in non-fibrotic areas is a predictor of a non-UIP pathological diagnosis. Moreover, when we analysed the diagnoses of these patients with significant mosaic attenuation only in non-fibrotic areas, we found that, even in patients with UIP pathologic diagnosis, this finding was associated with diagnoses other than IPF, corresponding to conditions in which mosaic attenuation is described as characteristic^4–7,13–15^, like HP or CTD-ILD. The finding of mosaic attenuation, when associated with fibrotic areas, probably reflects areas with expanded lobules to compensate the loss of air space and elasticity caused by adjacent fibrosis. It would thus not suggest a condition different from IPF. However, when mosaic attenuation is located in non-fibrotic areas, it could be related to air trapping caused by peribronchial granulomas in HP or follicular bronchiolitis associated with some CTD-ILDs.

Hochhegger et al.^18^ analysed air trapping and detailed its location in the upper lobes. Their results showed that the involvement of upper lobes was suggestive of diagnoses other than IPF, which was the case in 33.3% of the sample, compared to 3.9% of patients with IPF. We did not perform analyses by lung zones, but if we compare our results to those reported by these authors^18^, they may reflect similar findings. In IPF, fibrosis is usually predominant in lower fields, so mosaic attenuation in these areas would correspond to what we describe as “within fibrotic areas”. In contrast, mosaic attenuation in upper fields would be associated with areas with less fibrosis, which we classify as “in non-fibrotic areas”.

Regarding the other findings suggestive of another diagnosis, our results are similar, since most patients have some of them despite presenting a UIP pathologic diagnosis. For example, in our series, the absence of basal predominance is comparable in both UIP and non-UIP pathologic diagnosis. In the case of ground-glass, it is highly prevalent in both the UIP and non-UIP pathologic diagnosis, but it frequently appears within fibrotic areas in UIP, while in non-UIP it is more commonly in non-fibrotic areas. It is known that ground-glass found within areas of reticulation or with radiological signs of fibrosis probably translates to histological fibrosis and should not be considered a finding that excludes the diagnosis of UIP^1,2^.

When analysing the correlation between the pathological diagnosis and the radiological patterns, we found that most patients with a radiological pattern of probable UIP or indeterminate for UIP have UIP pathologic diagnosis, despite the presence of CT findings that are described as suggestive of another diagnosis. These results are in accordance with the development of radiological criteria in the latest guidelines, driven by the results of some studies reporting that the probability of having UIP pathologic diagnosis in non-typical patterns was elevated. Series that included only patients with IPF^8,20^ showed that more than 90% of patients with a radiological pattern defined as probable UIP had UIP pathologic diagnosis. In other cohorts more similar to those found in real clinical practice^21–23^, this proportion stood at 60 to 70%. Chung et al.^17^ reported UIP pathologic diagnosis in 89.6% of patients with HRCT indicative of UIP, in 81.6% of patients with probable UIP, and in 60.0% of patients with inconsistent with UIP. Few studies have been published applying the 2018 updates^1,2^. Fukihara et al.^24^ found that 82.6% of patients with a pattern of probable UIP had UIP pathologic diagnosis. A Japanese series^25^ analysed 27 patients with a pattern indeterminate for UIP, observing UIP/probable UIP pathologic diagnosis in 7 (25.9%). A French series^26^ reclassified patients with possible UIP based on the updated criteria, observing UIP pathologic diagnosis in 31/34 (91.2%) of those classified as probable UIP and in 5/7 (71.4%) of those as indeterminate for UIP.

Despite the current tendency in ILD to consider the progression of the fibrosis as the criteria for initiation of antifibrotic therapy, independently of the diagnosis (under the evolving concept of progressive fibrosing ILD)^27,28^, it will always be important to reach an etiological diagnosis in order to eventually take a different therapeutic approach. In this sense, the characteristics of mosaic attenuation mentioned above could be valuable to suggest either HP, which would prompt the inclusion of antigen avoidance as an important therapeutic measure, or alternatively CTD-ILD, which would include the consideration of treatment lines derived from anti-inflammatory or immunosuppressive therapy.

The main limitations of our study include the small number of patients, especially with UIP and probable UIP patterns, in whom lung biopsy is generally avoided, along with the retrospective application of the new radiological criteria. Moreover, the need for pathological confirmation as a reference standard represents a selection bias in itself, since only atypical cases should be pathologically confirmed, as demonstrated by the high percentage of indeterminate and non-UIP patterns. For this reason, the mean age of our population is low for patients with IPF, since biopsies are generally avoided in older patients. In any case, pathological confirmation adds value to the multidisciplinary diagnosis as the reference standard, thus increasing the diagnostic significance of the radiological findings described. Another limitation could be the observer subjectivity inherent to the evaluation of mosaic attenuation, as its definition varies across studies, and it can be influenced by the readers’ criteria. However, evaluation of its location in relation to fibrotic areas does not seem to be a limitation, as shown by the adequate interobserver correlation (κ = 0.610) that was even better than for the overall finding of mosaic attenuation (κ = 0.541). Finally, our study took place in a single centre, and readers also belonged to a single institution; however, ours is a reference hospital and included patients referred from other hospitals.

## Conclusions

In conclusion, findings suggestive of another diagnosis different from UIP such as mosaic attenuation are frequent in those patients with UIP pathologic diagnosis, so its mere presence does not appear sufficient to rule out UIP. However, in our series of pathologically confirmed ILD, the differential consideration of location of the mosaic attenuation in non-fibrotic areas was a predictor of non-UIP pathologic diagnosis and was associated with other diagnoses different from UIP, mostly CTD-ILD and HP. If confirmed in larger series, this finding could constitute a valuable tool for improving the interpretation of radiological patterns.

## Supplementary Information

Supplementary Information.
