# Author Correction: Mosaic attenuation in non-fibrotic areas as a predictor of non-usual interstitial pneumonia pathologic diagnosis

**DOI:** 10.1038/s41598-022-13314-x

**Published:** 2022-06-03

**Authors:** Ignacio Gayá García‑Manso, Juan Arenas‑Jiménez, Raquel García‑Sevila, Sandra Ruiz‑Alcaraz, Marina Sirera‑Matilla, Elena García‑Garrigós, María Ángeles Martínez‑García, Luis Hernández‑Blasco

**Affiliations:** 1 Department of Pulmonology, ISABIAL, Hospital General Universitario Dr. Balmis, Pintor Baeza, 11, 03010 Alicante, Spain; 2 Department of Radiology, ISABIAL, Hospital General Universitario Dr. Balmis, Alicante, Spain; 3 Department of Pulmonology, ISABIAL, Hospital General Universitario de Elche, Alicante, Spain; 4 Department of Clinical Medicine, UMH, Alicante, Spain

Correction to: *Scientific Reports* 10.1038/s41598-022-10750-7, published online 04 May 2022

The original version of this Article contained an error in the Abstract.

“If confirmed in larger series, this finding could constitute a valuable tool for improving the interpretation of radiological.”

now reads:

“If confirmed in larger series, this finding could constitute a valuable tool for improving the interpretation of radiological patterns.”

The original Article has been corrected.

