# Supplementary material for: Mosaic attenuation in non-fibrotic areas as a predictor of non-usual interstitial pneumonia pathologic diagnosis
Source: Sci Rep. 2022 May 4;12:7289. doi: 10.1038/s41598-022-10750-7 (PMC9068629; doi:10.1038/s41598-022-10750-7)
Supplement: Supplementary file 1 — Supplementary Information. [file 41598_2022_10750_MOESM1_ESM.pdf]

Title:

Mosaic attenuation in non-fibrotic areas as a predictor of non-usual interstitial pneumonia pathologic diagnosis

Authors:

- Ignacio **Gayá García-Manso**

Department of Pulmonology, Hospital General Universitario Dr. Balmis, ISABIAL, Alicante, Spain. ORCID 0000-0001-7228-061X nachogaya@gmail.com

- Juan **Arenas Jiménez**

Department of Radiology, Hospital General Universitario Dr. Balmis, ISABIAL, Alicante, Spain. j.arenasjimenez@gmail.com

- Raquel **García Sevila**

Department of Pulmonology, Hospital General Universitario Dr. Balmis, ISABIAL, Alicante, Spain. ORCID 0000-002-4218-793X raquelgsevila@gmail.com

- Sandra **Ruiz Alcaraz**

Department of Pulmonology, Hospital General Universitario de Elche, ISABIAL, Alicante, Spain. ruizalcarazsandra@gmail.com

- Marina **Sirera Matilla**

Department of Radiology, Hospital General Universitario Dr. Balmis, ISABIAL, Alicante, Spain. marinasirera@gmail.com

- Elena **García Garrigós**

Department of Radiology, Hospital General Universitario Dr. Balmis, ISABIAL, Alicante, Spain. piolelena@hotmail.com

- María Ángeles **Martínez García**

Department of Pulmonology, Hospital General Universitario Dr. Balmis, ISABIAL, Alicante, Spain. mangelesmtnez92@gmail.com

- Luis **Hernández Blasco**

Department of Pulmonology, Hospital General Universitario Dr. Balmis, ISABIAL. Department of Clinical Medicine. UMH. Alicante, Spain. ORCID 0000-0001-7484-2452 lhernandez@umh.es

Corresponding author:

Name: Ignacio Gayá García-Manso

Address: Pintor Baeza, 11, 03010 Alicante, Spain

Phone number: +34600228239 Email address: nachogaya@gmail.com

SUPPLEMENTARY MATERIAL

Exclusion criteria

We excluded patients with ILD who had undergone surgical lung biopsy but had other non-fibrosing diseases or a clear and known etiology, in which there was no clinical or radiological suspicion of IPF. For that reason, of the 113 patients who met our inclusion criteria, we excluded 30 in whom the radiological analysis led to a clear diagnosis other than IPF (e.g. lymphangioleiomyomatosis, histiocytosis) or who presented other diseases such as granulomatous conditions (sarcoidosis, pneumoconiosis) with a characteristic nodular radiological pattern not suggestive of fibrosing interstitial lung disease. These diagnoses are detailed in **Figure 2**. Nevertheless, we undertook HRCT readings for these cases, and both radiologists classified them as showing a non-UIP pattern with an alternative diagnosis. We also excluded patients with transbronchial or cryobiopsy specimens and those with surgical specimens obtained for reasons other than the diagnostic process for ILD (primarily resections for neoplasms).

Radiological evaluation

The CT scans were performed using different equipment from the various referring centres, so they had a wide range of technical characteristics. We included only the evaluations of an acceptable quality, without excessive noise or respiratory artefacts. Minimum technical characteristics were slices with a thickness of 1.5 mm or less, with lung window and high spatial reconstruction filter, and volumetric acquisition without spacing between reconstructions. In case of other phases, we read only inspiratory scans acquired in the supine position.

A rigorous process was established before reading, with two training sessions in which the three radiologists read more than 30 cases not included in the study in order to set fixed criteria to assess the radiologic findings and their quantification. We evaluated the chest CT images, noting the presence or absence of reticulation, traction bronchiectasis, honeycombing, ground-glass opacities, mosaic attenuation, emphysema, cysts, and consolidation. We considered mosaic attenuation to be the presence of pulmonary lobular or more extensive areas of lung with diminished attenuation compared to normal lung. For mosaic attenuation and the ground-glass opacities, these were described as alterations located within fibrotic areas (surrounded or next to lung tissue with reticulation, distortion, traction bronchiectasis, or honeycombing, without involving healthy lung tissue) or alternatively as only in non-fibrotic areas (when they were only surrounded by normal lung tissue). We quantified the overall extent of the disease, visually estimating the percentage of pathological lung, and for each finding we estimated the percentage of pathological lung that it represented, quantifying the extension in intervals of 5% in both cases. For mosaic attenuation, we recorded the number of lobes in which it was visible, considering the lingula as a lobe. For the analysis, we considered mosaic attenuation to be present when it was described by readers in at least one lobe, and it was considered significant when it affected three or more, as regarded in the description of findings suggestive of another diagnosis in previous guidelines [3]. The discrepancies in the quantification between the two radiologists were resolved by taking the average values of each observation. The distribution was analysed in both the axial (peribronchovascular, subpleural, or peripheral predominance) and the zonal (basal predominance) axes. To classify the radiological patterns, we used the criteria from the most recent ATS/ERS/JRS/ALAT guidelines [1].

Supplementary Table S.1. Interobserver agreement for classification of radiological patterns

Radiologist 1 (JAJ)	*Radiologist 2 (MSM)*			
	**UIP**	**Probable UIP**	**Indeterminate for UIP**	**Non-UIP**
**UIP**	9	1	1	0
**Probable UIP**	2	8	0	1
**Indeterminate for UIP**	1	5	8	0
**Non-UIP**	0	3	6	38

Data presented as n. UIP: usual interstitial pneumonia.

Supplementary Table S2. Relation between other radiological findings and radiological patterns on CT scan

	UIP (n=10)	*Probable UIP (n=11)*	*Indeterminate for UIP (n=15)*	*Non-UIP (n=47)*	*P value*
Reticulation	10 (100.0)	11 (100.0)	15 (100.0)	36 (76.6)	**0.021**
Extent of reticulation, %	90.0 (75.0-95.0)	85.0 (75.0-90.0)	85.0 (75.0-90.0)	55.0 (5.0-70.0)	**<0.001**
Traction bronchiectasis	10 (100.0)	11 (100.0)	15 (100.0)	31 (66.0)	**0.002**
Extent of traction bronchiectasis	67.5 (38.8-73.1)	30.0 (15.0-50.0)	25.0 (10.0-50.0)	10.0 (0.0-30.0)	**<0.001**
Honeycombing	10 (100.0)	2 (18.2)	10 (66.7)	3 (6.4)	**<0.001**
Extent of honeycombing, %	41.3 (20.0-64.4)	0.0 (0.0-5.0)	10.0 (0.0-50.0)	0.0 (0.0-0.0)	**<0.001**
Cysts	1 (10.0)	0 (0.0)	3 (20.0)	9 (19.1)	0.402
Emphysema	0 (0.0)	2 (18.2)	5 (33.3)	10 (21.3)	0.246
Consolidation	0 (0.0)	0 (0.0)	1 (6.7)	11 (23.4)	0.062
Extent of consolidation, %	0.0 (0.0-0.0)	0.0 (0.0-0.0)	0.0 (0.0-0.0)	0.0 (0.0-0.0)	0.059
Nodules	0 (0.0)	0 (0.0)	0 (0.0)	13 (27.7)	**0.008**
Lymph nodes	0 (0.0)	0 (0.0)	1 (6.7)	7 (14.9)	0.274
Overall extent of fibrosis, %	76.3 (70.0-85.0)	60.0 (35.0-75.0)	72.5 (65.0-80.0)	72.5 (65.0-80.0)	0.104
Peripheral predominance	10 (100.0)	11 (100.0)	12 (80.0)	24 (51.1)	**0.001**
Peribronchovascular predominance	2 (20.0)	1 (9.1)	4 (26.7)	29 (61.7)	**0.001**
Basal predominance	9 (90.0)	11 (100.0)	8 (53.3)	21 (44.7)	**0.001**

Data are presented as n (%) or mean ± SD or median (interquartile range). UIP: usual interstitial pneumonia. In bold statistically significant differences.

Supplementary Table S.3. Multidisciplinary diagnoses, radiological and pathologic patterns

Radiological pattern	Pathologic pattern	Multidisciplinary diagnosis
10 UIP	10 UIP	9 IPF
		1 CTD-ILD (Sjögren’s syndrome)
11 Probable UIP	9 UIP	9 IPF
	1 Bronchiolocentric interstitial pneumonia	1 Idiopathic bronchiolocentric interstitial pneumonia
	1 Lymphocytic interstitial pneumonia	1 Lymphocytic interstitial pneumonia associated to MCTD
15 Indeterminate for UIP	13 UIP	12 IPF
		1 HP
	2 NSIP	1 Idiopathic NSIP
		1 IPAF
47 Non-UIP	23 UIP	15 IPF
		4 CTD-ILD (2 Antisynthetase syndrome, 1 RA, 1 MCTD)
		3 HP
		1 IPAF
	7 NSIP	4 Idiopathic NSIP
		2 IPAF
		1 CTD-ILD (Antisynthetase syndrome)
	5 Unclassifiable fibrosis	2 Unclassifiable ILD
		2 CTD-ILD (1 AS, 1 UCTD)
		1 IPF
	4 Granulomatous disease	4 HP
	2 Bronchiolocentric interstitial pneumonia	2 Idiopathic bronchiolocentric interstitial pneumonia
	2 Follicular bronchiolitis	2 CTD-ILD (1 RA, 1 SLE)
	1 Lymphocytic interstitial pneumonia	1 GLILD
	1 GLILD	1 GLILD
	1 SRIF	1 SRIF
	1 Desquamative interstitial pneumonia	1 Desquamative interstitial pneumonia

UIP: usual interstitial pneumonia. IPF: idiopathic pulmonary fibrosis. CTD-ILD: connective tissue disease-associated interstitial lung disease. MCTD: mixed connective tissue disease. NSIP: nonspecific interstitial pneumonia. HP: hypersensitivity pneumonitis. IPAF: interstitial pneumonia with autoimmune features. RA: rheumatoid arthritis. AS: ankylosing spondylitis. UCDT: undifferentiated connective tissue disease. SLE: systemic lupus erythematosus. GLILD: granulomatous lymphocytic interstitial lung disease. SRIF: smoking-related interstitial fibrosis.
